# Precision management of medullary thyroid carcinoma: a dynamic framework integrating biomarkers, genotyping, and risk stratification

**DOI:** 10.3389/fendo.2026.1794118

**Published:** 2026-03-05

**Authors:** Chengzheng Jia, Shaohua Guo, Kehui Wu, Yaoqi Wang, Shuai Yang, Lei Wang, Tianyu Chen, Xianying Meng

**Affiliations:** 1 Department of Thyroid Surgery, General Surgery Center, The First Hospital of Jilin University, Changchun, China; 2 Department of Hepatobiliary and Pancreatic Medicine, The First Hospital of Jilin University, Changchun, China; 3 Department of Urology II, The First Hospital of Jilin University, Changchun, China; 4 Department of Urology I, The First Hospital of Jilin University, Changchun, China

**Keywords:** calcitonin, carcinoembryonic antigen, desmoplastic stromal reaction, medullary thyroid carcinoma, multigene panel, precision surgery, RET, targeted therapy

## Abstract

Medullary thyroid carcinoma (MTC) is a heterogeneous neuroendocrine malignancy in which outcomes are shaped by tumor burden, locoregional spread, and molecular context. Precision management therefore requires explicit separation of hereditary MTC driven by germline RET variants from presumed sporadic disease, and a structured integration of serum biomarkers, imaging, pathology, and genotype. This review synthesizes actionable evidence on calcitonin (Ctn) and carcinoembryonic antigen (CEA) baseline values and kinetics, universal germline RET testing, and tumor somatic profiling in advanced or progressive disease, and highlights desmoplastic stromal reaction (DSR) as an underused postoperative risk modifier in sporadic MTC. We propose a clinician-facing three-panel workflow: Panel A standardizes initial evaluation and mandates germline RET testing for all patients; Panel B outlines genotype- and staging-informed surgery and surveillance for hereditary disease, including pediatric carriers; and Panel C provides a staged approach for sporadic MTC in which imaging directs compartment selection and early postoperative DSR and biochemical response tailor surveillance intensity and thresholds for re-staging and re-intervention. By aligning decision nodes with real-world scenarios and using consistent surgical terminology, this framework offers a testable blueprint for precision surgery, surveillance stratification, and genotype-directed systemic therapy.

## 1 Introduction

Medullary thyroid carcinoma (MTC) accounts for a small proportion of thyroid malignancies but contributes disproportionately to thyroid cancer-related morbidity and mortality because of early lymphatic spread and limited sensitivity to radioiodine (1–3). Approximately one quarter of cases are hereditary, most commonly within multiple endocrine neoplasia type 2 (MEN2) driven by germline RET variants; the remainder are clinically sporadic, frequently harboring somatic RET or RAS alterations (4–7). Because these entities differ in biology, age of onset, and clinical pathways, precision management must avoid category errors that conflate hereditary and sporadic disease.

Calcitonin (Ctn) and carcinoembryonic antigen (CEA) remain indispensable for diagnosis and longitudinal monitoring, yet biomarkers alone do not define surgical compartments or systemic therapy selection for individual patients (8–11). Contemporary precision management increasingly depends on integrating biomarkers with mandatory molecular classification, high-quality imaging, and pathology-based risk modifiers. In particular, desmoplastic stromal reaction (DSR) in sporadic MTC has emerged as a clinically relevant correlate of locoregional aggressiveness and nodal involvement, but is rarely incorporated into decision pathways (12–15). Accordingly, this review emphasizes clear separation of hereditary versus sporadic scenarios, incorporation of DSR and other histopathologic modifiers, and a stepwise workflow that can be prospectively evaluated.

This narrative review was informed by searches of PubMed/MEDLINE and Embase for English-language studies, guidelines, and clinical trials on medullary thyroid carcinoma, calcitonin/CEA kinetics, hereditary MEN2/RET, somatic RET/RAS alterations, desmoplastic stromal reaction, neck dissection strategies, and targeted therapies. Search terms combined controlled vocabulary and keywords (e.g., “medullary thyroid carcinoma”, “calcitonin doubling time”, “RET”, “RAS”, “desmoplastic reaction”, “neck dissection”, “selpercatinib”, “pralsetinib”, “vandetanib”, “cabozantinib”). We prioritized practice guidelines, prospective trials, and high-quality surgical series; additional records were identified by screening reference lists. The aim is to synthesize clinically actionable principles rather than perform a formal meta-analysis.

## 2 Biomarkers, genomics, and histopathology

### 2.1 Traditional tumor biomarkers: enduring roles and limitations

Basal serum Ctn is the most sensitive marker of tumor burden in MTC and supports diagnosis, perioperative planning, and postoperative surveillance (4, 5, 8, 16). Sex-specific thresholds have been proposed to optimize diagnostic accuracy, with recent large-scale studies suggesting cutoffs of approximately 17–32 pg/mL for men and 7–23 pg/mL for women depending on population and assay platform (17, 18). CEA provides complementary information, particularly in tumors with discordant Ctn levels and clinical behavior; elevated CEA levels correlate with tumor burden and may signal dedifferentiation (8). For longitudinal risk assessment, biomarker kinetics (including doubling time) and the trajectory of change over time are often more informative than isolated absolute values (19–22). Calcitonin doubling time (CDT) less than 6 months is associated with approximately 8% 10-year survival, whereas CDT greater than 24 months corresponds to survival exceeding 90% (20, 21).

However, Ctn and CEA should not be treated as stand-alone determinants of surgical extent. Both markers are affected by assay variability and comorbidities (e.g., renal dysfunction), and preoperative levels correlate with nodal disease only at a population level rather than reliably localizing occult metastases in an individual patient. Approximately 5-10% of MTCs are non-secretory and show no meaningful rise in serum Ctn (23). Procalcitonin has emerged as an alternative biomarker with comparable performance and greater analytical stability (24, 25). Chromogranin A is a pan-neuroendocrine marker that may be elevated in MTC but has limited diagnostic sensitivity (26). Biomarker-informed decisions should therefore be interpreted alongside structured neck ultrasound, cross-sectional imaging when indicated, and molecular classification (hereditary *vs* sporadic).

### 2.2 Multigene testing and molecular classification

Molecular classification is the cornerstone of precision management in MTC. Germline RET testing should be performed in all patients with MTC, irrespective of apparent sporadic presentation, because the result directly determines family counseling, cascade screening, and the timing of intervention in relatives (4, 5, 27, 28). The RET proto-oncogene encodes a receptor tyrosine kinase, and activating mutations are found in virtually all hereditary MTC and approximately 50% of sporadic cases (6, 29). The 2015 ATA guidelines classify hereditary MTC into three risk categories based on RET genotype: highest risk (ATA-HST; M918T), high risk (ATA-H; codon 634), and moderate risk (ATA-MOD; other pathogenic variants) (4). This genotype-based stratification informs surgical timing and broader clinical decision-making (30).

In advanced, recurrent, or rapidly progressive disease, tumor somatic profiling (RET point mutations/fusions, RAS, and other actionable alterations when present) is clinically actionable for selecting targeted therapy and trial eligibility (31–35). RAS mutations (predominantly HRAS) are the second most common driver in MTC and are found in approximately 70% of RET-wild-type sporadic tumors (7, 36). RAS-mutant MTC tends to present at an older age and exhibit less aggressive behavior than RET-mutant disease (36, 37). TERT promoter mutations, though uncommon (~5-10%), are strongly associated with aggressive tumor behavior (38). Recently, NF1 inactivation has been identified as a driver in approximately 11% of RET/RAS-negative MTC (39, 40). ALK fusions, though rare (<2%), represent actionable therapeutic targets (41, 42).

Hereditary MTC (germline RET) and sporadic MTC (germline RET-negative) represent distinct clinical entities with different decision drivers. Accordingly, management algorithms should separate genotype-driven timing and prevention strategies used for RET carriers from staging- and imaging-led surgical decision-making typical of sporadic disease. The workflow proposed below separates these pathways while maintaining shared entry points and terminology.

In clinical practice, for RET/RAS-negative MTC, multigene panels that include NF1 and ALK are recommended primarily in advanced or metastatic disease where identifying actionable alterations can directly inform targeted therapy selection (e.g., ALK inhibitors for ALK-fusion-positive tumors) or clinical trial eligibility, rather than as part of routine initial workup for all MTC patients.

### 2.3 Desmoplastic stromal reaction and histopathologic risk modifiers in sporadic MTC

Desmoplastic stromal reaction (DSR) refers to a prominent fibrotic stromal response at the invasive tumor front and reflects tumor-stroma crosstalk that can accompany infiltrative growth (12). In sporadic MTC, DSR has been associated with increased likelihood of lymph node metastasis and locally invasive behavior in several surgical series, making it a clinically relevant risk modifier that complements tumor size and biochemical burden (12–15). In one landmark study, all DSR-negative tumors were node-negative and all patients achieved biochemical cure, whereas 36% of DSR-positive tumors had nodal metastases (12). Subsequent studies have confirmed DSR positivity as an independent risk factor for nodal involvement (13–15).

DSR is typically assessed on routine hematoxylin-eosin sections from the primary surgical specimen (and is not reliably available preoperatively). This makes DSR most useful as an early postoperative decision point, ideally reported in a standardized “present/absent” format together with other adverse features (extrathyroidal extension, lymphovascular invasion, high mitotic activity/Ki-67 index, tumor necrosis, and nodal burden) (22, 43). When combined with early biochemical response (e.g., time to calcitonin normalization and whether postoperative Ctn becomes undetectable or remains persistently elevated), DSR can refine the intensity of surveillance, lower the threshold for early re-staging imaging, and support consideration of additional compartment-oriented surgery in selected patients with biochemically persistent, anatomically localizable disease (44–47).

Importantly, the postoperative availability of DSR is aligned with its intended clinical role: it refines surveillance intensity and re-staging thresholds at a decision point where preoperative factors have already guided initial surgery. This positioning resolves a common gap in algorithms that attempt to use postoperative features as if they were available preoperatively.

## 3 Application of genetic testing in MTC

**Figure 1:**
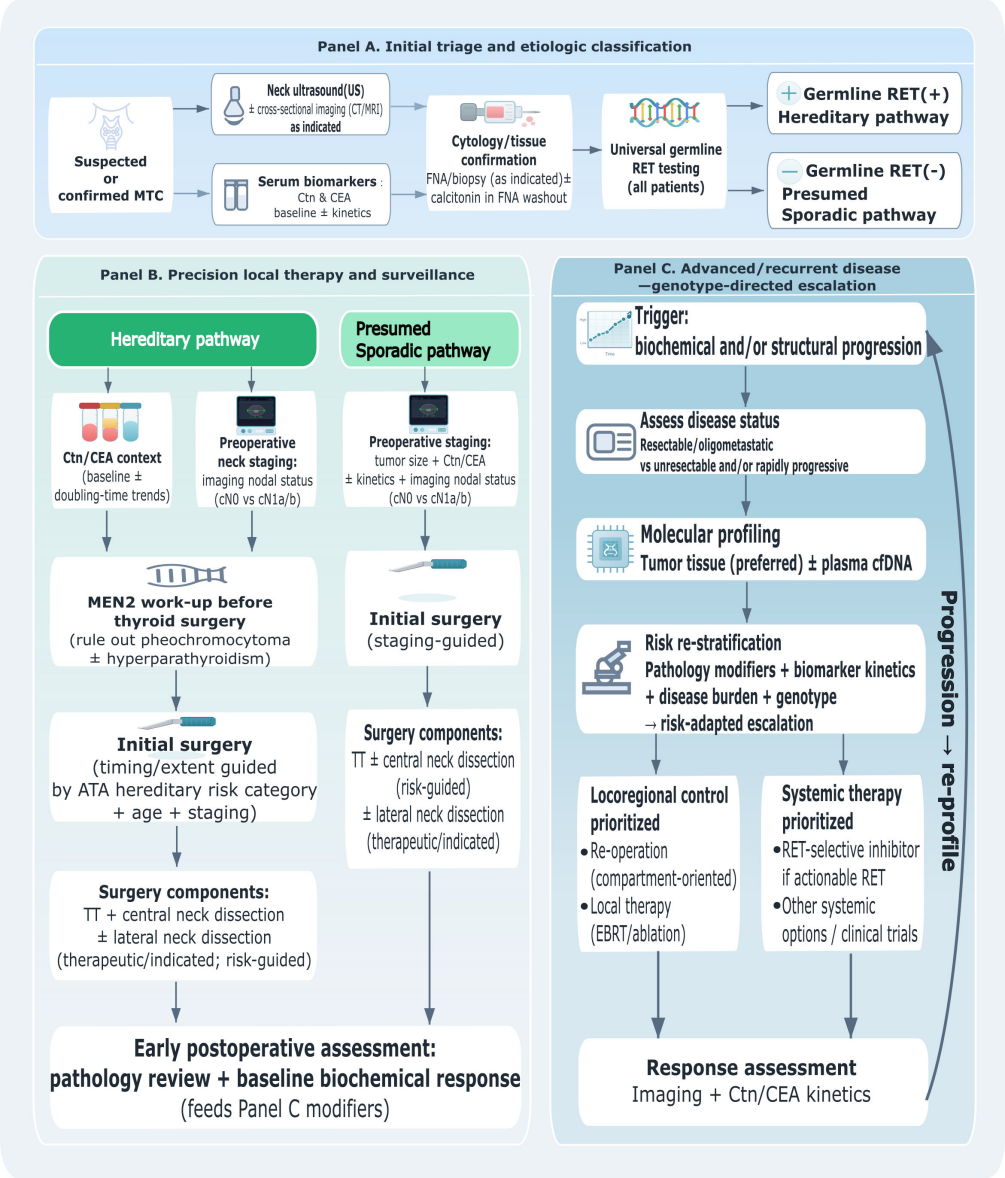
Precision-medicine workflow integrating biomarkers and genetic testing in medullary thyroid carcinoma. MTC, medullary thyroid carcinoma; Ctn, calcitonin; CEA, carcinoembryonic antigen; RET, rearranged during transfection; US, ultrasound; CT, computed tomography; MRI, magnetic resonance imaging; FNA, fine-needle aspiration; cfDNA, cell-free DNA; MEN2, multiple endocrine neoplasia type 2; ATA, American Thyroid Association; TT, total thyroidectomy; EBRT, external beam radiotherapy; DT, doubling time; cN0, clinically node-negative; cN1a/b, clinically node-positive central (N1a)/lateral (N1b) compartments.

The proposed precision-management workflow is summarized in **Figures 1A-C**. It uses universal entry steps for all patients, and then applies distinct pathways for hereditary versus sporadic MTC to avoid category errors and to improve real-world applicability (3, 48).

### 3.1 Panel A: initial evaluation and mandatory classification (all patients)

Confirm diagnosis and establish a baseline: measure basal Ctn and CEA; perform comprehensive neck ultrasound with structured reporting of central and lateral compartments; and obtain cross-sectional imaging when clinically indicated (e.g., bulky primary tumor, suspected mediastinal/retropharyngeal nodes, symptoms, or biochemical burden out of proportion to neck findings) (4, 5, 49, 50). Ultrasound features suspicious for malignancy correlate with advanced TNM stage and aggressive behavior (49). Perform universal germline RET testing at diagnosis and initiate genetic counseling pathways. Until germline results are known, the index patient should be managed as potentially hereditary for counseling and family-risk purposes, while surgical decisions remain grounded in clinical staging and imaging (4, 5, 27). Completeness of RET testing has improved substantially over recent decades but remains suboptimal in some regions (51). Plan primary surgery using an anatomic-first principle: total thyroidectomy is generally recommended for clinically apparent MTC (4, 5). Central compartment dissection is typically performed with therapeutic intent based on the assessed risk of nodal involvement and local practice patterns. Lateral neck dissection should be therapeutic (performed when there is imaging and/or cytologic evidence of lateral nodal disease), or risk-guided elective in carefully selected settings where the probability of occult metastasis is high and the compartment at risk is clearly defined; it should not be labeled “prophylactic” in this context (52).

### 3.2 Panel B: hereditary MTC (germline RET) — genotype- and biomarker-informed management

In germline RET carriers, genotype (ATA risk category) and age primarily determine the timing of thyroidectomy, particularly in childhood, whereas Ctn/CEA and imaging refine the extent of nodal surgery (4, 30). In truly prophylactic thyroidectomy (asymptomatic carrier, normal biomarkers, no suspicious nodes), routine lateral neck dissection is not indicated; central dissection may also be individualized (53). For carriers with biochemical evidence of disease or suspicious nodes, nodal management becomes therapeutic. Preoperative basal Ctn, structured ultrasound, and (when applicable) cross-sectional imaging guide whether central compartment dissection alone is sufficient or whether lateral compartment dissection is warranted. In practice, proven central nodal metastasis and/or marked biochemical burden increases the pretest probability of occult lateral disease; in such settings, a risk-guided elective lateral neck dissection may be considered and should be distinguished from prophylactic thyroidectomy.

Clinical example: a young child with an ATA highest-risk RET variant and a low but detectable basal Ctn should be managed within the genotype-driven pathway (timely genotype-guided thyroidectomy, with nodal surgery tailored to imaging and biomarkers), rather than forced into an adult sporadic biomarker-only model.

### 3.3 Panel C: sporadic MTC (germline RET-negative) — integrating biomarkers, imaging, and early postoperative pathology

After germline RET negativity is confirmed, sporadic MTC should be managed through staged integration of anatomic extent, biochemical burden/kinetics, and early postoperative modifiers (48). Preoperatively, tumor size and basal biomarkers can inform the likelihood of nodal disease (62), but imaging remains the primary determinant of compartment selection for lateral neck dissection (4, 5, 49, 50).

Postoperatively, two variables are particularly useful for refining risk: (1) early biochemical response (whether Ctn becomes undetectable or remains persistently elevated) and (2) histopathologic aggressiveness, especially DSR (12–15, 46). A patient with a moderate-sized sporadic tumor and intermediate biomarker levels but absent DSR and prompt biochemical remission may be managed with standard surveillance (47). Conversely, the combination of DSR and biochemical persistence should prompt early re-staging (cross-sectional imaging ± functional imaging, depending on availability) and consideration of additional compartment-oriented surgery when disease is anatomically localized.

Clinical example: a 20-mm sporadic MTC with basal Ctn around the low-to-moderate hundreds and no DSR on pathology fits the sporadic pathway; DSR is treated as a postoperative risk modifier rather than a preoperative requirement, thereby resolving a common gap in overly unified algorithms.

## 4 Practical considerations and implementation

### 4.1 Integration with systemic therapy (advanced or progressive disease)

For unresectable, recurrent, or metastatic disease, multigene testing becomes directly actionable (31, 54). Molecular profiling should be performed when systemic therapy is being considered, preferentially on tumor tissue; circulating tumor DNA can be considered when tissue is limited or when a rapid, minimally invasive assessment is needed (55, 56). When actionable RET alterations are detected, highly selective RET inhibitors (selpercatinib, pralsetinib) can provide durable disease control with objective response rates near 70% (31–33). When no actionable RET alteration is identified or selective agents are unavailable, multi-kinase inhibitors (vandetanib, cabozantinib, anlotinib) and/or clinical trials remain appropriate options (34, 35, 57).

Precision management in this setting requires coordinated interpretation of genotype, the pace of progression (biomarker kinetics and imaging), and symptom burden to determine the timing and sequencing of systemic therapy, local control measures for oligoprogression, and trial referral (54, 58). To translate the above principles into practice, **Table 1** summarizes the key decision drivers that distinguish hereditary (germline RET+) from presumed sporadic (germline RET–) medullary thyroid carcinoma across the care continuum.

**Table 1:** Key decision drivers in hereditary versus presumed sporadic MTC (summary for clinical use).

Domain	Hereditary MTC (germline RET+)	Presumed sporadic MTC (germline RET−)
Primary decision driver(s)	Genotype (ATA risk category) and age; biomarkers/imaging refine nodal extent (4, 5).	Anatomic extent and imaging; biomarkers support risk context; postoperative pathology (DSR) refines risk (12–15).
Timing of thyroidectomy	Genotype- and age-guided (especially in childhood); biomarker rise supports manifest disease (30, 53).	Typically at diagnosis of clinically apparent MTC; timing driven by staging and operability.
Central compartment management	Individualized; therapeutic intent when disease is suspected/confirmed; not routine in truly prophylactic carrier surgery.	Often performed with therapeutic intent based on staging and practice patterns; guided by imaging and intraoperative findings.
Lateral neck management	Therapeutic when cN1b; risk-guided elective only in selected high-probability settings (biochemical burden + central nodal disease) with clear compartment target (52).	Therapeutic when cN1b; selective risk-guided elective in cN0 only when strongly indicated; imaging defines compartments.
Key postoperative modifiers	Biochemical response and kinetics; nodal burden; adverse pathology when present (20–22).	Early biochemical response plus DSR and other adverse features (grade/Ki-67, ETE, LVI, nodal burden) (44, 45).
When to perform tumor somatic profiling	Primarily for advanced/recurrent disease or systemic therapy decisions (despite germline driver).	At recurrence/metastasis or before systemic therapy; tissue preferred, ctDNA when tissue limited (55, 56).
Systemic therapy selection	RET-directed therapy when actionable alterations are present; otherwise MKIs/clinical trials; integrate pace and symptoms (31–35).	RET inhibitors for RET-altered disease; MKIs/clinical trials otherwise; integrate imaging and kinetics (54, 58).

MTC, medullary thyroid carcinoma; RET, rearranged during transfection; ATA, American Thyroid Association; DSR, desmoplastic stromal reaction; ETE, extrathyroidal extension; LVI, lymphovascular invasion; ctDNA, circulating tumor DNA; MKI, multikinase inhibitor; cN0, clinically node-negative; cN1b, clinically node-positive lateral neck nodes.

While ATA and NCCN guidelines provide essential foundational recommendations, the present framework advances clinical practice by: (1) explicitly separating hereditary and sporadic pathways to prevent category errors in surgical planning; (2) incorporating DSR as a postoperative risk modifier with specific decision thresholds; (3) providing a testable, visual workflow with standardized surgical terminology; and (4) integrating early postoperative pathology with biochemical kinetics for dynamic risk re-stratification—features not systematically addressed in current guideline formats. **Table 2** illustrates how the integrated framework applies to representative clinical scenarios across hereditary and sporadic settings.

**Table 2:** Clinical scenarios illustrating genotype- and pathology-informed decision-making.

Scenario	Patient profile	Traditional approach	Integrated framework approach
A. Pediatric hereditary MTC	8-year-old, ATA-HST (M918T), basal Ctn 45 pg/mL, cN0	May delay surgery awaiting higher Ctn or apply adult sporadic thresholds	Genotype-driven pathway: timely thyroidectomy guided by ATA risk category + age; central dissection individualized
B. Sporadic MTC, low-risk	45-year-old, germline RET-negative, 15-mm tumor, Ctn 120 pg/mL, DSR-negative, postop Ctn undetectable	Standard surveillance regardless of pathology	Standard surveillance confirmed; DSR absence + biochemical cure supports lower re-staging threshold
C. Sporadic MTC, high-risk	52-year-old, germline RET-negative, 22-mm tumor, Ctn 350 pg/mL, DSR-positive, postop Ctn 25 pg/mL	Surveillance based on Ctn kinetics alone	Early cross-sectional re-staging prompted by DSR + biochemical persistence; consideration of compartment-oriented re-intervention

MTC, medullary thyroid carcinoma; ATA-HST, ATA highest-risk; Ctn, calcitonin; cN0, clinically node-negative; DSR, desmoplastic stromal reaction; postop, postoperative.

These drivers provide a pragmatic scaffold for the risk-adapted workflow detailed in the subsequent sections.

### 4.2 Surgical terminology and practical clarifications

The term prophylactic should be reserved for thyroidectomy performed in an asymptomatic germline RET carrier before clinically manifest MTC (4, 53). In contrast, neck dissection performed because metastatic disease is confirmed or strongly suspected (even when not clinically palpable) is more accurately described as therapeutic, or as risk-guided elective when performed in a clinically node-negative patient with compelling evidence that occult disease is likely confined to a definable compartment (52). Using precise terminology improves clinical coherence and reduces inappropriate extrapolation between hereditary and sporadic settings.

### 4.3 Implementation considerations in resource-variable settings

Access to comprehensive genetic testing, standardized pathology reporting (including DSR), and highly selective targeted agents varies across health systems and can influence the feasibility of precision pathways (51, 54, 58). Practical implementation priorities include routine germline RET testing with streamlined cascade screening, standardized reporting of key histopathologic modifiers (DSR, lymphovascular invasion, extrathyroidal extension, grade), and multidisciplinary decision-making that explicitly separates hereditary and sporadic scenarios while applying shared anatomic principles of compartment-oriented surgery (48). Where access to advanced imaging or selective targeted agents is limited, emphasis should be placed on high-quality ultrasound staging, consistent biomarker kinetics reporting, and referral networks for molecular testing and clinical trials.

## 5 Future directions

Prospective validation is required to determine whether integrated frameworks improve outcomes compared with traditional marker-driven approaches. Areas of active development include circulating tumor DNA and other minimal residual disease assays (55, 56), and harmonization of biomarker assays across centers. Immune checkpoint inhibitors are being explored in combination with targeted agents, though comprehensive immune profiling suggests MTC has a relatively “cold” tumor microenvironment (59, 60). Artificial intelligence-driven approaches may enhance diagnostic accuracy and prognostic precision (61). Importantly, proposed algorithms should be evaluated separately in hereditary and sporadic cohorts to avoid category errors and to ensure clinical applicability.

## 6 Conclusion

Precision management of MTC requires mandatory molecular classification and clinically coherent pathways. By separating hereditary (germline RET) and sporadic disease while integrating biomarkers, imaging, and early postoperative pathology—particularly desmoplastic stromal reaction—clinicians can move beyond repetitive marker descriptions toward actionable, testable decision-making that supports precision surgery, surveillance stratification, and genotype-directed systemic therapy.
